# Spontaneous enterocutaneous fistula in a patient with femoral hernia: a case report

**DOI:** 10.1186/s12893-021-01439-1

**Published:** 2021-12-25

**Authors:** Anup Chalise, Ashish Prasad Rajbhandari, Lok Bahadur Kathayat, Rabin Koirala

**Affiliations:** Nepal Medical College and Teaching Hospital, Attarkhel, Kathmandu, Nepal

**Keywords:** Enterocutaneous fistula, Femoral hernia, Incarcerated hernia, Spontaneous fistula, Case report [MeSH]

## Abstract

**Background:**

Enterocutaneous fistula commonly occurs in the post-operative setting. However, a handful of cases have been reported to occur secondary to strangulation of hernia, mostly femoral due to the narrow femoral ring through which this type of hernia passes through.

**Case presentation:**

We encountered a case of spontaneous fecal fistula, which occurred in the setting of an incarcerated femoral hernia. The patient did not develop peritonism, or obstruction, throughout the course of the disease. The hernia ruptured on day 7 of incarceration. Exploratory laparotomy under epidural anesthesia revealed a femoral hernia with ileum as content, arising approximately 20 cm from the ileocecal junction. Reduction of the contents was done, and a resection performed along with repair of the hernia.

**Conclusion:**

As very few literature describe the formation of spontaneous fecal fistula, we discuss the presentation in this report.

## Background

Femoral hernia is one of the types of groin hernia, with approximately 20% incidence amongst all abdominal hernias in females and 5% in males [1]. The hernia passes through a narrow femoral ring, which is why they are more prone to strangulation. Similarly, enterocutaneous fistula, or fecal fistula, occur more commonly in the post-operative period following mesh repair of complicated hernias [2]. Post operative cases account for up to 85% of these causes and occur more in the emergency surgical setting [3]. Other common causes may include radiation, inflammatory bowel disease, diverticular disease, and acute appendicitis [3]. Only a handful of these reported cases have been secondary to femoral hernia. In our literature review, we found that there has been only one previous report of a female patient with femoral hernia presenting as enterocutaneous fistula [4].

## Case presentation

A 72-year-old multiparous female presented to the Emergency Department at Nepal Medical College and Teaching Hospital, with a history of on and off groin swelling since the past 2 months, which had become irreducible in the last 10 days prior to presentation. She had no history of nausea, vomiting, fever, colicky abdominal pain, or abdominal distention. However, 3 days prior to her visit, she noticed that the skin around the swelling had become red and had started to ooze purulent material. The next day, there was discharge of fecal matter from the lump.

**Fig. 1:**
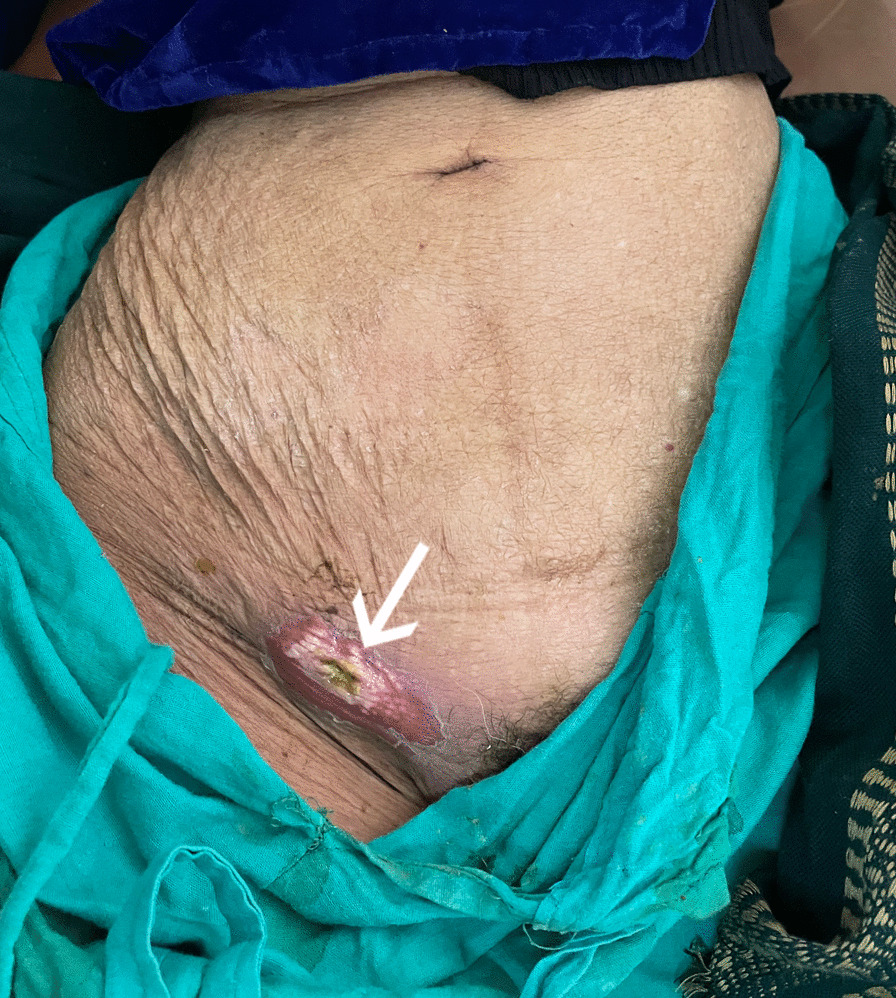
Site of fistula opening in right groin

At presentation, her vitals were stable and there was no evidence of peritonitis on physical examination. She did not have any features of obstruction, however, there was an enterocutaneous fistula in the right groin (Fig. 1). There were no swellings in the left groin, or in the umbilicus. The fistula was of low output type and was noted to be discharging fecal material. The patient also did not have evidence of sepsis or require inotropic support during the duration of treatment.

**Fig. 2:**
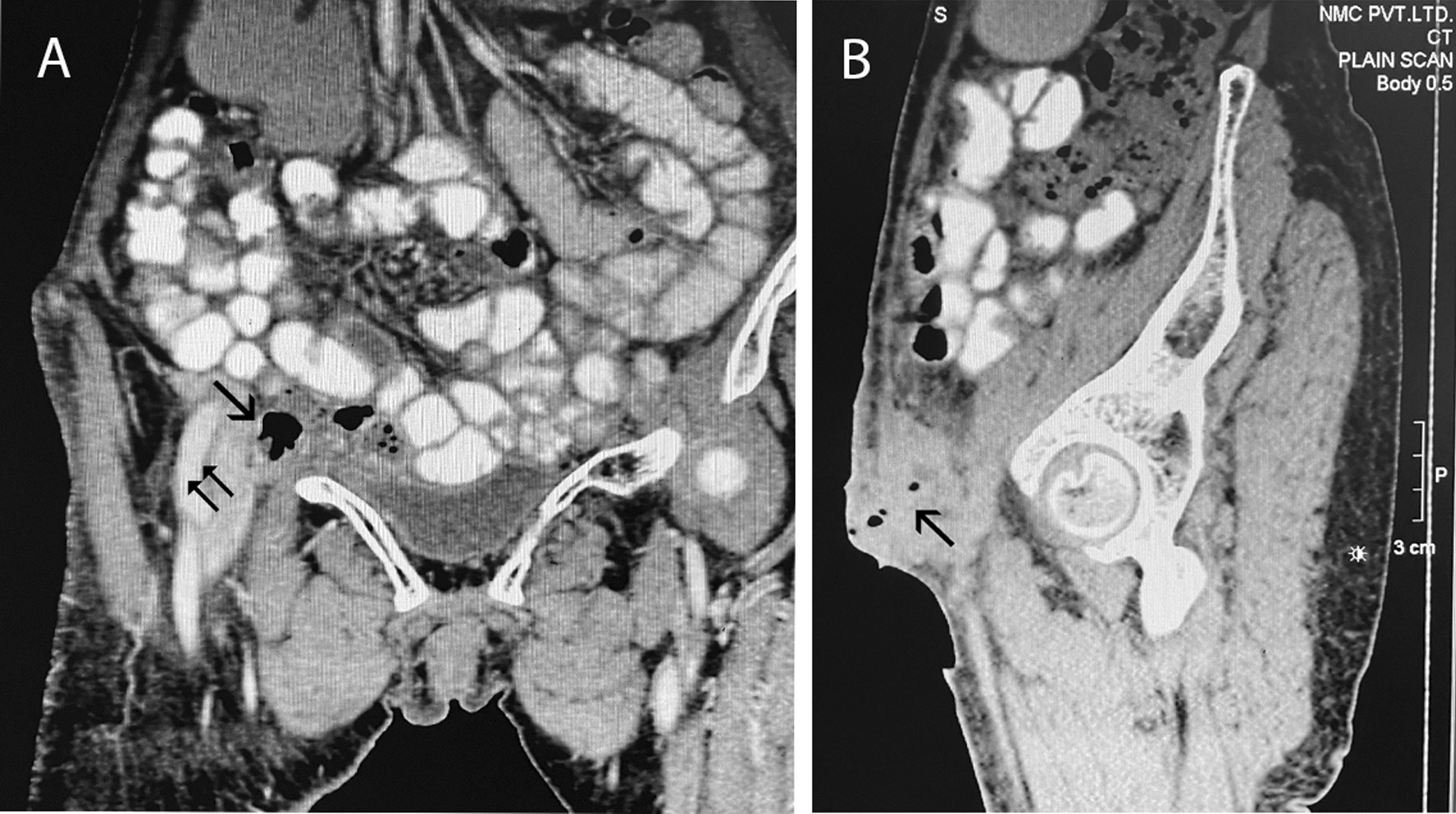
CT scan findings showing location of fistulation and femoral hernia

**Fig. 3:**
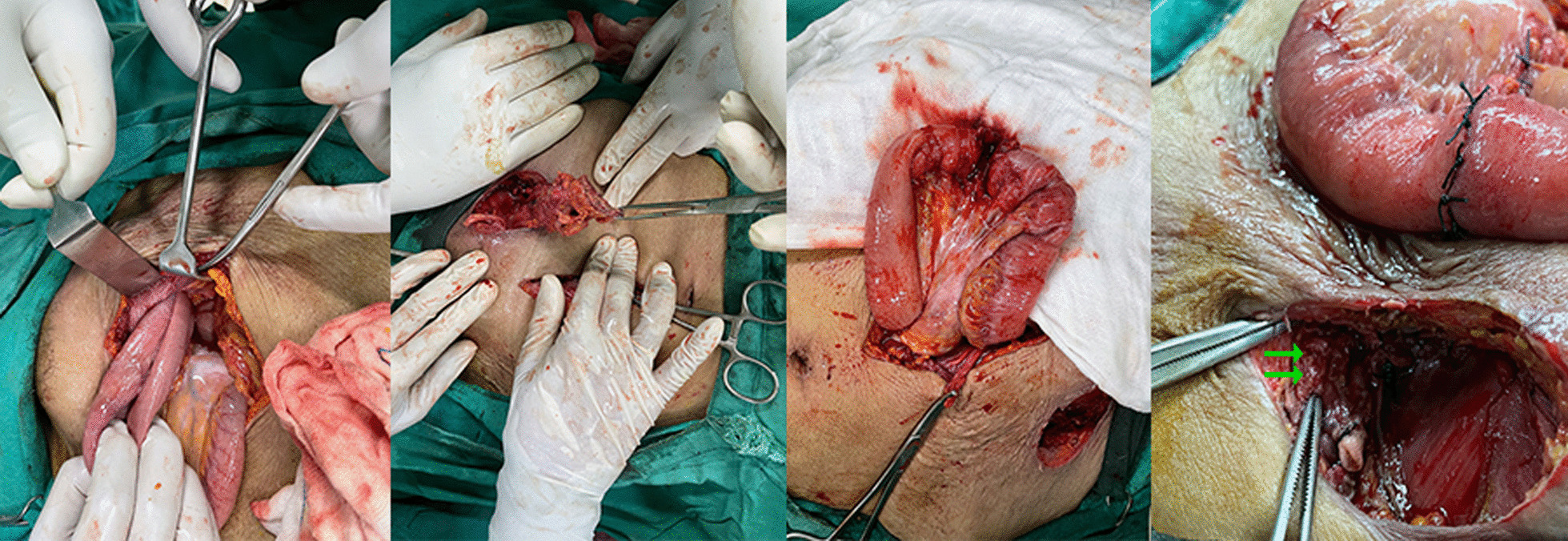
Intra-op photos showing (left-to-right): Loop of herniated bowel; Excised external component of fistula; Bowel loop pre-repair; Bowel loop and relation of defect (double green arrows) post repair

The patient was admitted, resuscitated, and a contrast enhanced computed tomography scan was done, which showed findings consistent with enterocutaneous fistula coming out through the femoral ring (Fig. 2). The patient then underwent exploration. This was done under epidural anesthesia as the patient had crepitations over bilateral lung fields. A lower midline incision was given to help reduce the contents of the hernia, and the enterocutaneous fistula was excised. Although most femoral hernias are Richter’s type, our patient had a bowel loop that had incarcerated, but only the apical portion of the loop had become gangrenous and ultimately perforated as an enterocutaneous fistula. The hernial orifice admitted approximately 2 fingers (~ 2.5 cm), but with difficulty. The herniated bowel loop was approximately 20 cm from the terminal ileum, so a resection and anastomosis of approximately 10 cm of the bowel was done, with 5 cm on each size of the perforation, and a drain placed. A primary repair of the femoral ring was performed with polypropylene sutures from both the groin incision and intraperitoneally (as mesh was contraindicated in this patient), and the skin was kept open (Fig. 3). The post-operative period was uneventful, and the patient was then discharged on post-op day 5, with the skin incision closed with polyamide sutures, and the drain removed on the day of discharge. Currently the patient is doing well, and the wounds have healed without surgical site infection, or any complications requiring readmission.

## Discussion and conclusions

Most of the femoral hernia have a segment of bowel herniating through them, which is eponymously termed as Richter’s hernia. This type of hernia will rapidly progress to gangrene as the constricting ring exerts pressure on the bowel wall, compromising blood flow. Features of intestinal obstruction are typically absent, especially when less than two-thirds of the circumference of the bowel is involved, which leads to late diagnosis or bowel ischemia [5]. Our patient had a complete loop of bowel in the femoral ring, rather than the usual Richter’s type hernia usually reported in such patients.

An enterocutaneous fistula (ECF), as the name describes, forms when there is an abnormal communication between the gastrointestinal tract and the skin (or wound, when there is one). The most common cause of ECF is iatrogenic, i.e., it follows a visceral surgery [6, 7]. Only about a quarter of these cases may occur spontaneously and do so most commonly in the setting of inflammatory bowel disease like Crohn’s [7]. Other common causes of spontaneous ECF include malignancy, appendicitis, tuberculosis, radiation exposure and mesenteric ischemia [5, 8]. The conversion of an incarcerated or strangulated hernia to an ECF leads to bowel decompression, like how a stoma would function. However, there is an increase in incidence of septic complications and mortality, especially with high-output fistulae (> 500 mL/24 h) [3].

Femoral hernia commonly presents with obstruction and strangulation, formation of ECF in these patients is a rare entity, like the presentation of our patient [6]. Further, our patient’s hernia was similar to a Richter’s type, signifying that only a part of the bowel wall was trapped in the hernial sac, even though an entire loop had herniated (possibly due to the patulous hernial orifice), which led to the formation of ECF [9]. This explains why the patient did not have signs of obstruction or peritonitis, and why the patient’s fistula output was low with fecal material as content.

Treatment of ECF comprises of using the SNAP guideline, which has been proven effective in treating such cases [7]. The first step is resuscitation, followed by the identification of sepsis. This is followed by identifying the nutrition status and addressing these issues. If the patient is well-nourished and the effluent from the fistula well controlled, like in our case, this is followed by imaging to delineate the anatomy. The last step is a definite procedure to close the fistula and address the underlying issues. Our patient was managed according to this guideline, and she showed rapid improvement and an uneventful recovery.

Although a surgical repair of hernia in an elective setting utilizes synthetic mesh placement to reinforce surgical closure of the defect, this is contraindicated in patients with an enterocutaneous fistula, such as our case. Hence, a mesh was not placed in this patient [10, 11].

Most patients presenting in our setup are poor, lack the appropriate level of knowledge required for timely consults and neglect their health care till an emergency arises. These factors may play a role in formation of such fistulae, even when in this modern era such complications can easily be prevented by timely interventions. However, even delayed presentations can be managed, like in our case, by adhering to a set of fixed protocols like the SNAP guideline.

## Data Availability

Not applicable.

## Abbreviations

CT: Computed tomography
ECF: Entero-cutaneous fistula
SNAP: Sepsis, nutrition, anatomy, procedure

## References

[1] Nikolopoulos I, Oderuth E, Ntakomyti E, Kald B (2014). Intestinal obstruction due to bilateral strangulated femoral hernias. Case Rep Surg.

[2] Sistla S, Reddy R, Dharanipragada K, Jagdish S (2008). Enterocutaneous fistula due to mesh fixation in the repair of lateral incisional hernia: a case report. Cases J.

[3] Berry S, Fischer J (1996). Classification and pathophysiology of enterocutaneous fistulas. Surg Clin N Am.

[4] Bostanci M, Ozel I, Bozkurt B, Soylu S, Turan M (2015). Spontaneous enterocutaneous fistula: a rare presentation of incarcerated femoral hernia. Eurasian J Em Med.

[5] Ahi K, Moudgil A, Aggarwal K, Sharma C, Singh K (2015). A rare case of spontaneous inguinal faecal fistula as a complication of incarcerated Richter’s hernia with brief review of literature. BMC Surg.

[6] Kumar A, Pahwa HS, Pandey A, Kumar S (2012). Spontaneous enterocutaneous fistula due to femoral hernia. Case Rep.

[7] Gribovskaja-Rupp I, Melton G (2016). Enterocutaneous fistula: proven strategies and updates. Clin Colon Rectal Surg.

[8] Shah M, Wani M (2017). Spontaneous tubercular enterocutaneous fistula. Saudi J Med Med Sci.

[9] Skandalakis P, Zoras O, Skandalakis J, Mirilas P (2006). Richter hernia: surgical anatomy and technique of repair. Am Surg.

[10] Birolini C, de Miranda J, Tanaka E, Utiyama E, Rasslan S, Birolini D (2019). The use of synthetic mesh in contaminated and infected abdominal wall repairs: challenging the dogma—a long-term prospective clinical trial. Hernia.

[11] Ramaswamy A. Use of synthetic mesh in the infected field—a SAGES Wiki Article. SAGES. 2021. https://www.sages.org/wiki/use-synthetic-mesh-infected-field/. Accessed 4 Dec 2021.

